# The Influence of Tunnel Parameters and Graft Inclination Angle on Clinical and Radiological Outcome at Long-term Follow-up after Arthroscopic Anterior Cruciate Ligament Reconstruction

**DOI:** 10.1055/s-0044-1785493

**Published:** 2024-04-10

**Authors:** Thatchinamoorthy Santhamoorthy, Anish Anto Xavier, Kaliaperumal Krun, Dharamveer Kumar Dubey

**Affiliations:** 1 Departamento de Ortopedia, Indira Gandhi Government General Hospital and Postgraduate Institute, Puducherry, Índia; 2 Departamento de Ortopedia, Indira Gandhi Medical College and Research Institute, Puducherry, Índia

**Keywords:** anterior cruciate ligament reconstruction, knee joint, osteoarthritis

## Abstract

**Objective** To study the influence of various tunnel parameters and graft inclination angle (GIA) on the clinical and radiological outcome after anterior cruciate ligament reconstruction (ACLR) at long-term follow-up.

**Methods** In this retrospective study, 80 patients with isolated anterior cruciate ligament (ACL) injury treated by single bundle ACLR using bone patellar tendon bone (BPTB) and hamstring (HT) autografts were evaluated clinically and radiologically at their long-term follow-up. The study population were divided into two groups based on ideal and nonideal tunnel parameters as well as ideal and nonideal GIA. The various tunnel parameters and GIA were interpreted with clinical and radiological outcome at long-term follow-up.

**Results** Eighty patients, 36 (45%) using BPTB and 44 (55%) using HT autografts, were available to complete the study. Patients with ideal coronal tibial tunnel angle (CTTA) and coronal femoral tunnel angle (CFTA) show superior clinical outcome (pivot shift test) than nonideal CTTA and CFTA, which was found to be statistically significant ( *p* -value < 0.038 and 0.024, respectively). Similarly, patients with ideal coronal tibial tunnel position (CTTP) show superior clinical outcome (International Knee Documentation Committee - IKDC objective) over nonideal CTTP ( *p* -value < 0.017). All other tunnel parameters and GIA were not found to have influence on clinical outcome. None of the tunnel parameters have influenced osteoarthritis (OA) change. There was no progression of OA change in the study population at long-term follow-up after ACLR.

**Conclusion** Ideal coronal tunnel parameters produced a better clinical outcome at long-term follow-up after ACLR. There was no progression of OA change at long-term follow-up after isolated ACLR.

## Introduction

Arthroscopic anterior cruciate ligament reconstruction (ACLR) is the standard of treatment for anterior cruciate ligament (ACL) insufficiency. The goal of ACLR is to restore normal knee anatomy and kinesiology, which will improve knee stability. Recent literature states that early osteoarthritis (OA) changes may be aborted in ACL injured knee by anatomical ACLR. 1 The tibial and femoral tunnel placements primarily are important in achieving knee stability. Improper femoral or tibial tunnel placement were the most commonly argued cause for failure of ACLR. 2 Cadaveric studies have shown that the location of the center of native ACL in the femur, on lateral radiograph, is present at a mean of 66% of the anterior edge of the Blumensaat line. On the tibial side, the center of native ACL is located at the junction of the anterior and middle third of the tibial plateau. 3 Supporting the cadaveric studies, recent clinical studies show that anatomical ACLR would result in better clinical outcome than non-anatomical reconstruction. 4 Traditional transtibial (TT) technique would cause more vertical orientation of graft, decreased rotational stability, and graft failure. 5 It was argued that anteromedial (AM) portal aids in more anatomical femoral tunnel placement compared to traditional TT portal. The grafts in anatomical ACLR lie in more horizontal position in coronal plane resulting in improved rotational stability and decreases the pivot shift phenomenon. 6 Though ideal tunnel parameters and GIA representing anatomical ACLR has been emphasized in many studies in the past, their influence on long-term outcome has not been validated. Further, a few authors are of the opinion that there is no correlation between tunnel position and long-term clinical outcome. 7 Also, recent studies show non-anatomical femoral tunnel placement prevails equally among TT as well as in AM portal techniques. 8 In spite of extensive literature studies on tunnel placement, the ideal tunnel placement has not been found in many patients due to some variables. The commonly used tunnel parameters include coronal femoral tunnel position (CFTP), sagittal femoral tunnel position (SFTP), coronal tibial tunnel position (CTTP), sagittal tibial tunnel position (STTP), coronal femoral tunnel angle (CFTA), sagittal femoral tunnel angle (SFTA), coronal tibial tunnel angle (CTTA), sagittal tibial tunnel angle (STTA), and graft inclination angle (GIA).

Hence, we proposed to study, retrospectively, the influence of various tunnel parameters and GIA on the clinical and radiological outcome after ACLR at long-term follow-up.

We hypothesize that:

1. Ideal coronal tunnel parameters would result in better clinical outcome.
2. Progression of knee osteoarthritis (OA) would be retarded by ACLR in isolated ACL injury.

## Materials and Methods

This retrospective study was conducted in a tertiary care center in South India. Institutional review board approval was obtained, and all patients in the study signed the written informed consent.

From January 2013 to august 2016, 80 patient's data were collected from hospital registry comprising 36 patients who underwent ACLR with bone-patellar tendon-bone (BPTB), and 44 patients with hamstring tendon (HT) autografts.

Inclusion criteria:

• Age > 18 years and < 45 years
• Sex: males and females
• Isolated ACL injury
• Willingness to participate in the study
Exclusion criteria:
• Associated meniscal and chondral injuries
• Multiligamentous injury
• Previous surgeries in ipsilateral limb or knee
• Concomitant fractures in ipsilateral or opposite limb
• Reinjury to operated knee
• Patients with generalized ligament laxity
• Patients not willing to participate in the study

## Surgical Procedure

All the patients in this study underwent arthroscopic ACLR by a single fellowship trained arthroscopic surgeon. AM portal technique was used in all cases for femoral tunnel creation. Patients received either BPTB or HT autograft. Both femoral and tibial side graft fixation was done using titanium interference screw (Nebula, India).

Postoperative rehabilitation:

**Table:** 

Week 1	Isometric quadriceps, ankle pumps, active straight leg raise (SLR) exercise with knee brace support.
Week 2	Previous exercise+ patellar mobilization and closed chain knee range of movement exercise restricting to 90 deg flexion.
Week 3–6	Previous exercise + progressive hamstring and quadriceps strengthening exercise.
Week 6–3 Months	Previous exercise + progressive knee flexion + proprioception and core strengthening exercise.
3–6 Months	Swimming, progressive squatting, and sports-specific agility training exercises.

### Radiological Evaluation

**Fig. 1:**
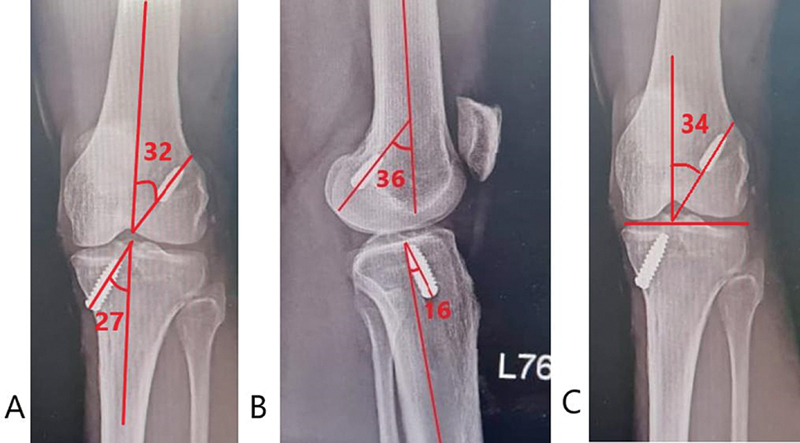
Radiological assessment of tunnel angles in postoperative x-rays. A) Coronal femoral and tibial tunnel angles. B) Sagittal femoral and tibial tunnel angles. C) Graft inclination angle.

**Fig. 2:**
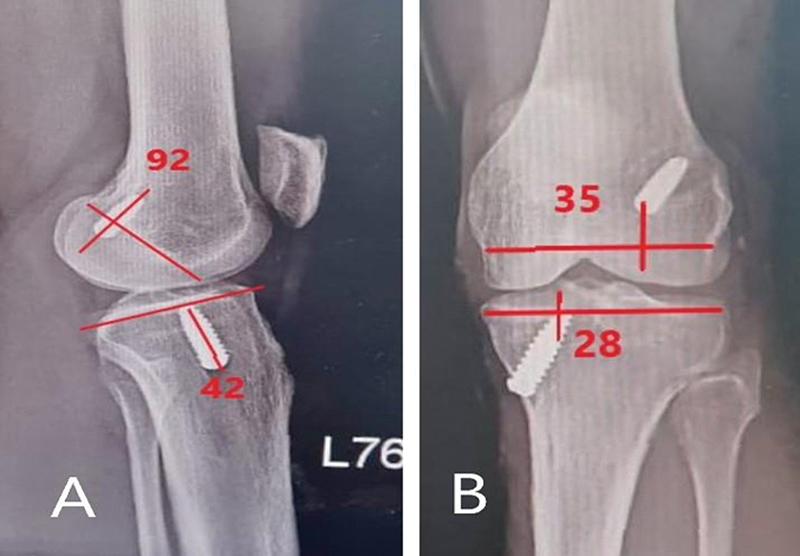
Radiological assessment of tunnel positions in post operative x-ray. A) Coronal femoral and tibial tunnel positions. B) Sagittal femoral and tibial tunnel positions.

Preoperative plain radiographs of injured knee were compared with postoperative x-rays. Standing AP view with 0 ^o^ knee flexion, PA view with 30 ^o^ knee flexion, and lateral view with 30 ^0^ knee flexion were taken to analyze. All radiograph images were interpreted in DICOM format in Picture Archiving and Communication Systems (PACS) (version 8.2). Various tunnel parameters, GIA, and OA change measurements were analyzed. The tunnel parameters and radiological measurements analyzed in the study were CFTA, SFTA, CTTA, STTA, CFTP, SFTP, CTTP, STTP, and GIA ( Figs. 1 and 2 ). For OA change analysis, we used the Kellegran and Lawrence (KL) score. 9 Radiological assessment was done by two persons (radiologists) with reliable inter and intra observer correlation.

### Clinical Evaluation

This includes International Knee Documentation Committee (IKDC)-subjective and objective, Lysholm scoring, single leg hops testing (SLHT), pivot shift test, anterior drawer test (ADT), and Lachman test (LT).

The study population was divided into two groups based on ideal vs nonideal tunnel parameters and ideal GIA vs nonideal GIA.

**Fig. 3:**
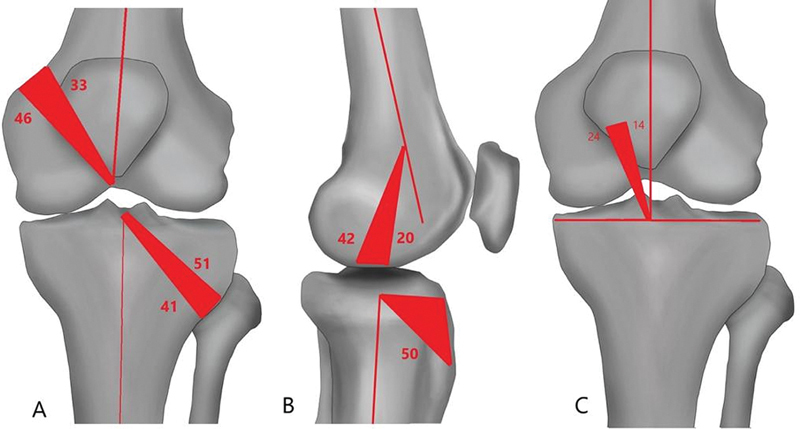
Schematic diagram showing ideal tunnel angle range. A) Ideal coronal femoral and tibial tunnel angle range. B) Ideal sagittal femoral and tibial tunnel angle range. C) Ideal graft inclination angle range.

**Fig. 4:**
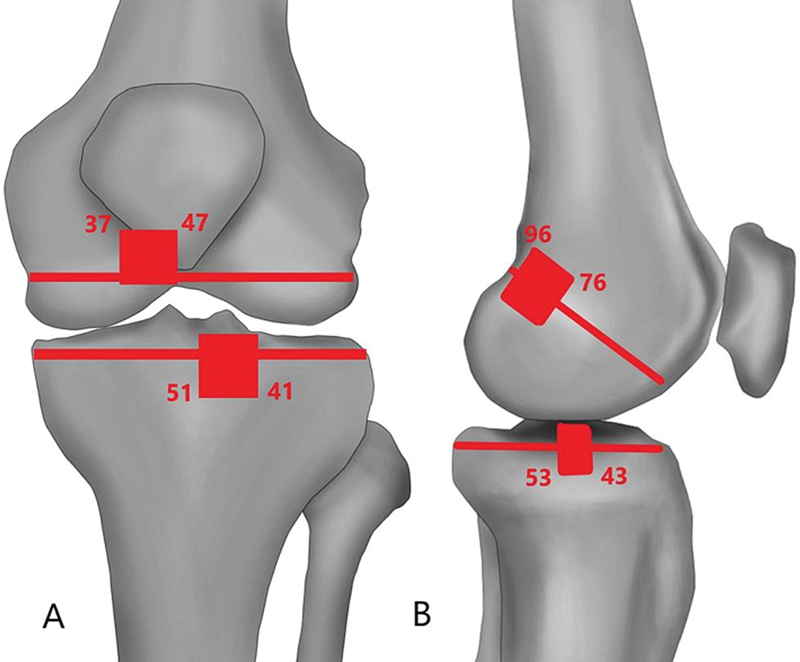
Schematic diagram showing ideal tunnel position range. A) Ideal coronal femoral and tibial tunnel position range. B) Ideal sagittal femoral and tibial tunnel position range.

Ideal tunnel parameters and ideal GIA were those patients in whom the tunnel measurements were within the reference range recommended by previous studies. 10 11 12 13 14 Nonideal tunnel parameters and nonideal GIA were those patients in whom the measurements were outside the reference range as recommended by previous studies ( Figs. 3 and 4 ). The Pivot shift test was dichotomized into two subgroups for statistical analysis. In one group grade 0, and another with grades 1, 2, and 3. Similarly, ADT and LT were dichotomized into two groups. In one group grade 1, and in another group grades 2 and 3. Likewise, IKDC (objective) and single leg hop test were dichotomized. In one group grades A and B, and another with grades C and D.

The clinical and radiological outcome were interpreted in both ideal and nonideal tunnel groups as well as in both ideal and nonideal GIA groups at long term follow-up.

## Statistical Analysis

The statistical analysis was done using IBM SPSS Statistics for Window version 26.0 (IBM Corp., Armonk, NY, USA). Descriptive data like mean, median, and standard deviation (SD) were entered as numbers and percentage. Analytical statistics were done using the chi-square test, Man-Whitney test and coupled t-test after dichotomization. *P* -value was significant if < 0.05.

## Results

**Fig. 5:**
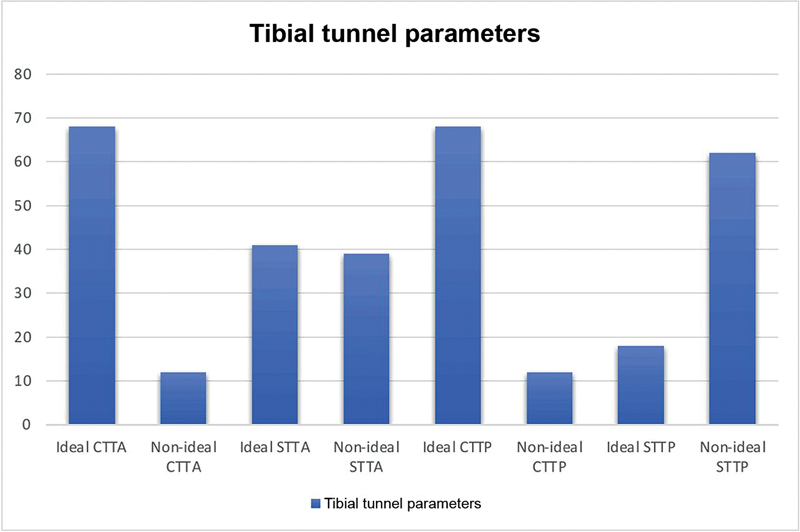
Distribution of ideal/nonideal tibial tunnel parameter among study population.

Eighty patients, comprising 36 (45%) using BPTB and 44 (55%) using HT autografts, were taken in the study. The mean (SD) age of the subjects was observed to be 31.25 (6.83). Around 34 (42.5%), 32 (40%), and 14 (17.5%) belonged to the age group 20 to 29 years, 30 to 39 years and 40 to 49 years, respectively. Male and female subjects accounted for 71 (88.8%) and 9 (11.3%), respectively. Road traffic accidents (34 [42.5%]), fall from one's own height (24 [30%]), and sports injury, (22 [27.5%]), were reported to be the different modes of injury among the subjects. The median (interquartile range - IQR) time from injury was found to be 90 (30–180) days. The mean (SD) follow-up time for the patients was 98.59 (+/-13.78) months. The distribution of ideal/nonideal tunnel parameters and ideal/nonideal GIA among the study subjects is shown in Figs. 5 6 7 .

**Fig. 6:**
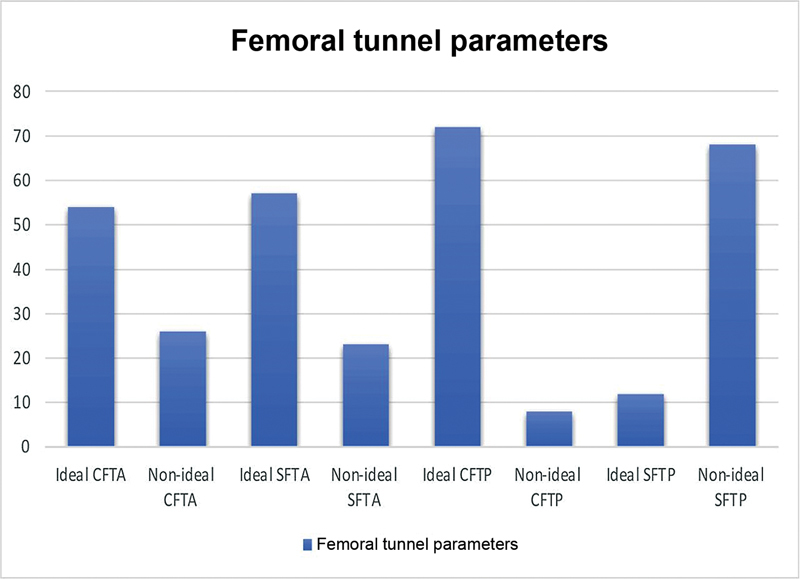
Distribution of ideal/nonideal femoral tunnel parameters among study population.

**Fig. 7:**
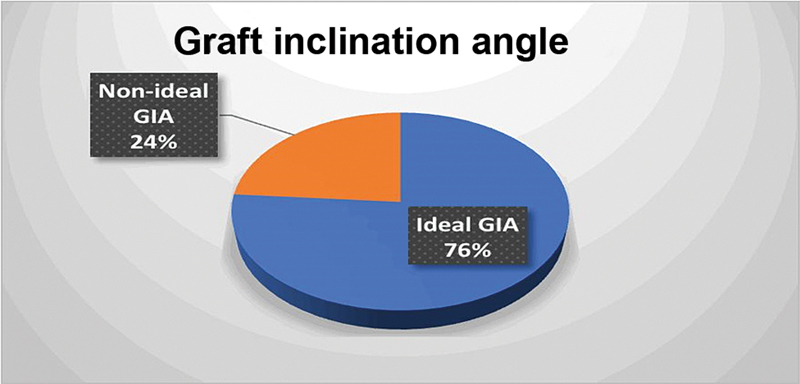
Distribution of ideal/nonideal GIA among study population.

The mean of ideal tunnel parameters and GIA of our study was compared with literature reference values, as shown in Table 1 .

**Table 1:** Comparison of Mean of the tunnel parameters and GIA of our study with Literature values

Tunnel parameters	Our study Mean (SD)	Reference value Mean (SD)	Literature
CFTA	35.13 (7.61)	39.5 (6.5)	Illingworth et al. (2011)
SFTA	23.85 (11.315)	31 (11)	Takeda et al. (2013)
CTTA	19.4 (8.824)	28 (18)	Kondo et al. (2007)
STTA	31.33 (7.202)	69.5 (19.5)	Kondo et al. (2007)
CFTP	41.76 (3.135)	42 (5)	Pinczewski et al. (2008)
SFTP	59.14 (16.834)	86 (10)	Pinczewski et al. (2008)
CTTP	44.86 (4.469)	46 (5)	Pinczewski ET AL. (2008)
STTP	37.98 (8.35)	48 (5)	Pinczewski et al. (2008)
GIA	15.81 (7.621)	19 (5)	Pinczewski et al. (2008)

Abbreviations: CFTA - coronal femoral tunnel angle; SFTA - sagital femoral tunnel angle; CTTA - coronal tibial tunnel angle; STTA - Sagital tibial tunnel angle; CFTP - coronal femoral tunnel position; SFTP - sagital femoral tunnel position; CTTP - coronal tibial tunnel position; STTP - sagital tibial tunnel position; GIA - graft inclination angle.

With regard to subjective clinical outcome, the mean values (SD) of preop and postop IKDC (subjective) were 49.15 (9.86) and 91.10 (6.97), respectively. Whereas the mean (SD) preop and postop Lysholm scores were found to be 38.30 (11.52) and 94.06 (4.81), respectively. There was no significant difference between ideal and nonideal tunnel parameters as well as GIA with regard to subjective clinical outcome.

The distribution of clinical and radiological outcome in the study subjects is shown in Table 2 . The current postoperative KL score remains the same as that of preoperative KL scores in the study population. The statistical association between various tunnel parameters and clinical outcome (objective) is shown in Table 3 . There was no significant difference between ideal and nonideal GIA on clinical (objective) and radiological outcome, as shown in Table 4 . Similarly, there was no significant association between other tunnel parameters on radiological outcome as shown in Table 5 .

**Table 2:** Distribution of clinical outcome and radiological outcome in the study subjects

Clinical objective outcome parameters	Preoperative N (%)	Current postoperative N (%)
**Pivot shift test**		
Grade 0	0 (0)	36 (45)
Grade 1	2 (2.5)	42 (52)
Grade 2	50 (62.5)	2 (2.5)
Grade 3	28 (35)	0(0)
**Objective IKDC score**		
A	0 (0)	48 (60)
B	0 (0)	31 (38.8)
C	74 (92.5)	1 (1.3)
D	6 (7.5)	0 (0)
**Single-leg hop test IKDC grade**		
A	0 (0)	62 (77.5)
B	0 (0)	15 (18.8)
C	0 (0)	3 (3.8)
**Anterior Drawers test**		
Grade 1	0 (0)	37 (46.3)
Grade 2	34 (42.5)	39 (48.8)
Grade 3	46 (57.5)	4 (5)
**Lachman test**		
Grade 1	0 (0)	36 (45)
Grade 2	30 (37.5)	42 (52.5)
Grade 3	50 (62.5)	2 (2.5)
**KL score**		
Grade 1	22 (27.5)	22 (27.5)
Grade 2	6 (7.5)	6 (7.5)
Normal	52 (65)	52 (65)
**IKDC (subjective)**		
Mean	49.499	91.067
Minimum	6.9	66.7
Maximum	71.3	98.9
Std Deviation	13.856	6.099
**Lysholm score**		
Mean	38.35	94.05
Minimum	2	80
Maximum	66	100
Standard deviation	11.584	4.846

Abbreviations: IKDC - International Knee Documentation Committee.

**Table 3:** Statistical association between various tunnel parameters and clinical outcome (objective) expressed as *p* -values

Ideal vs nonideal	SINGLE-LEG HOP TEST	IKDC (OBJ)	PIVOT	ADT	LT
CTTA	0.458	0.672	0.038*	0.363	0.766
STTA	0.527	0.302	0.514	0.666	0.514
CTTP	0.365	0.017*	0.378	0.730	0.801
STTP	0.062	0.062	0.127	0.080	0.095
CFTA	0.975	0.147	0.024*	0.332	0.737
SFTA	0.858	0.113	0.503	0.191	0.096
CFTP	0.556	0.737	0.294	0.331	0.294
SFTP	0.365	0.672	0.378	0.109	0.378

*- denotes *p* -value < 0.05.

Abbreviations: CFTA - coronal femoral tunnel angle; SFTA - sagital femoral tunnel angle; CTTA - coronal tibial tunnel angle; STTA - sagital tibial tunnel angle; CFTP - coronal femoral tunnel; position; SFTP - sagital femoral tunnel position; CTTP - coronal tibial tunnel position; STTP - sagital tibial tunnel position; GIA - graft inclination angle; ADT - Anterior Drawer Test; LT- Lachman test; IKDC - International Knee; Documentation Committee.

**Table 4:** Association between graft inclination angle and outcome

Ideal vs nonideal	SINGLE LEG HOP TEST	KL SCORE	IKDC	PIVOT	ADT	LT
GIA ( *p* -value)	0.325	0.672	0.574	0.196	0.066	0.068

Abbreviations: GIA- Graft inclination angle; KL SCORE - Kellgren and Lawrence Score; IKDC - International Knee; Documentation Committee; ADT - Anterior Drawer Test; LT- Lachman test.

**Table 5:** Association between tunnel parameters and radiological outcome

Ideal vs nonideal	CTTA	STTA	CTTP	STTP	CFTA	SFTA	CFTP	SFTP
KL SCORE ( *p* -value)	0.905	0.078	0.285	0.093	0.341	0.796	0.058	0.905

Abbreviations: KL SCORE - Kellgren and Lawrence Score; CFTA - coronal femoral tunnel angle; SFTA - sagital femoral tunnel angle; CTTA - coronal tibial tunnel angle; STTA - sagital tibial tunnel angle; CFTP - coronal femoral tunnel position; SFTP - sagital femoral tunnel position; CTTP - coronal tibial tunnel position; STTP - sagital tibial tunnel position.

Patients with ideal CTTA and CFTA show superior clinical outcome (pivot shift test) than nonideal CTTA and CFTA, and it is found to be statistically significant ( *p* -value < 0.038 and 0.024, respectively). Similarly, patients with ideal CTTP show superior clinical outcome (IKDC objective) over nonideal CTTP ( *p* -value < 0.017), as shown in Table 6 .

**Table 6:** Ideal coronal tunnel parameters showing statistaclly significant superior clinical outcome over non-ideal coronal tunnel parameters

Tunnel parameters		Clinical outcome (Pivot shift test)		Chi-square ( *p* -value)
		Gr-0	Gr-1, 2, 3	
**CTTA**	Ideal	31	29	0.038
	Nonideal	5	15	
**CFTA**	Ideal	29	25	0.024
	Nonideal	7	19	
		**Clinical outcome (IKDC Objective)**		
		**GR-A & B**	**GR-C & D**	
**CTTP**	Ideal	68	0	0.017
	Nonideal	11	1	

Abbreviations: CFTA - coronal femoral tunnel angle; CTTA - coronal tibial tunnel angle; CTTP - coronal tibial tunnel position; gr - grade.

## Discussion

The primary finding in our study is that patients with ideal CFTA and CTTA were associated with better rotational stability in the form of decreased pivot shift grade. This implies that ideal CFTA and CTTA, which would produce more oblique femoral and tibial tunnels, result in better rotational stability. Similarly, patients with ideal CTTP have better IKDC (objective) score. Other tunnel parameters and GIA were not found to have any significant impact on the clinical outcome.

Hence, our first hypothesis is proved to be correct as ideal coronal tunnel parameters produced better clinical outcome in the form of improved pivot shift grade and significantly better IKDC (objective) score. Similarly, our second hypothesis is also proved to be correct as there was no progression of knee OA after isolated ACLR in the study population on long-term follow up. From the present study, it is clear that accurate coronal placement of grafts, both on the femoral and tibial sides, is essential to get better long-term clinical outcome after ACLR.

According to various studies, placement of graft more vertically in the coronal plane would cause the graft to impinge on the lateral part of the posterior cruciate ligament (PCL) causing loss of flexion and decreased anterior stability resulting in poor clinical outcome. 5 11 15 16 A femoral tunnel placed more obliquely in the coronal plane is important for rotational stability of the knee. 10

Although it was believed that the anteromedial (AM) portal technique would aid in low position and more oblique coronal femoral tunnel angle, there is still controversy regarding its superiority over the TT technique. Ruhr-Wagner et al. 17 reported increased risk of revision surgeries with the AM portal technique compared to the TT technique. Similarly, Jaecker et al. 8 found in their study high rates of nonanatomic femoral and tibial tunnel positions in ACL revisions with both AM and TT femoral drilling techniques. In our study, too, we used only AM portal technique for femoral drilling in all cases. We did find nonanatomical tunnel positions and angles in many of our study population. This may be attributed to individual distal femoral anatomic variations such as narrow femoral notch, which may not allow for an obliquely drilled tunnel, which, in turn, can lead to anteriorly placed nonanatomical tunnel with a decreased CFTA, as postulated by Illingworth et al. 11

In our study, the mean CFTA was 35.13 ^0^ . Illingworth et al. 11 evaluated coronal femoral tunnel angle in 45 ^o^ knee flexion weight-bearing PA radiographs in postoperative ACLR and found that a CFTA of < 32.7 ^o^ is likely to have ACLR that falls outside an anatomic range. In our study, patients with nonideal femoral tunnel angles, < 32 ^o^ , presented increased pivot shift grading postoperatively. This contrasts with the studies by Sundemo et al. 7 and Moghtadaei et al., 18 in which they found no influence of CFTA on clinical or radiological outcome. In the current study, we found that ideal CFTA patients have significantly improved pivot grading compared to nonideal CFTA patients. In our study, the mean CTTA was 19.40 ^0^ . The ideal CTTA described in the literature was between 60 and 65 ^o^ (measured between the tibial plateau and the tunnel). 19 This will prevent PCL impingement and decrease anterior laxity. In our study, the CTTA was measured between the anatomical axis of the tibia and the tibial tunnel, as described by Kondo et al. 13 A similar measurement was done by Mightadaei et al. 18 In their study, CTTA did not influence the ACLR outcome. In our study, we found ideal CTTA patients had significantly better rotational stability than nonideal CTTA patients. According to Pinczewski et al. 10 and Topliss and Webb, 20 the tibial tunnel should be at 47% from the medial cortex across the tibial plateau in the coronal plane. They state that a more medial placement could cause impingement. In our study, the mean CTTP was 44.86 (+/- 4.46). Patients with ideal CTTP in our study had better IKDC (objective) score than those with nonideal CTTP.

Debnath et al. 21 did a radiological evaluation of the tunnel position in single-bundle ACLR in the Indian population and correlated with clinical outcome. They found that the “ideal clinical outcome” was significantly associated with placement of the femoral tunnel along the sagittal plane. They also recommend that the femoral tunnel should not be placed beyond the 85% mark along the Blumensat line from the anterior most point. Xu et al., 22 in a systemic review, found the mean position of the native femoral insertion was 28.4% (+/- 5.1%) from the posterior border when using the quadrant method. Sundemo et al. 7 reported mean SFTP by the quadrant method was 40% (+/- 6.4%) from posterior to anterior. In our study, the mean SFTP was 59.14 (+/-16.83). We found no influence of SFTP on the clinical or radiological outcome. According to Ristić et al., 14 the acceptable STTA was 50 to 89 ^o^ (avg 68 ^o^ ). These authors state that a significant deviation from these values may potentially lead to failure of the ACLR. In our study, the mean STTA was 31.33 ^o^ (+/- 7.20). There was no difference in outcome between ideal and nonideal STTA in our study.

Moisala et al. 23 stated that the optimal tibial tunnel location on the sagittal plane is between 32 and 37% of the length of the tibial plateau from the anterior corner for better clinical outcome. In our study, STTP was 37.98 (+/- 8.35). We could not find any impact of STTP on clinical outcome.

We analyzed the impact of various tunnel parameters and graft inclination angle on the outcome of ACLR using the two commonly used autografts. Only very few authors have done similar studies on tunnel parameters in the past using these two grafts with variable results. 7 24 25

Pinczewski et al. 10 and Struewer et al. 26 reported the progression of the OA in their study population after ACLR in the long term. Contrary to their findings, in our study there was no influence of tunnel parameters or GIA in the long-term OA changes in the study population. Our results were similar to those of Sundemo et al. ^7^ Surprisingly, in our study, we found that patients with preexisting OA also did not show worsening in the long term. This implies that more than tunnel parameters and GIA, other factors, like concomitant meniscal or chondral injuries, could contribute more for the progression of OA changes in the long term after ACLR.

## Limitations

Our study has a few limitations. We have performed the study only in isolated ACL patients. In order to have adequate study population inclusion of two types of autografts was unaviodable. Though this made the study population heterogenous, it did not influence the outcome. Being a retrospective study, a potential selection bias was unavoidable. We used only plain radiography for assessing tunnel parameters. The anteroposterior and pivot shift test grading were performed manually instead of with instrumented arthrometers.

The current study was unique in a way that we analyzed the influence of as many as nine tunnel-related parameters, which include both femoral and tibial tunnel positions, tunnel angles, and GIA, on clinical and radiological outcome in the long term, which makes ours distinguishable from other studies.

## Conclusion

In our study, patients with ideal coronal tunnel parameters showed significantly better rotational stability and clinical outcome compared with those with non-ideal coronal tunnel parameters at long-term follow-up after ACLR. Sagittal tunnel parameters and GIA did not have any significant influence on the radiological or clinical outcome after ACLR. None of the tunnel parameters or GIA had influence on OA changes in the long term. Future prospective studies comparing clinical and radiological long-term outcome following ACLR using three-dimensional computed tomography (3D CT) scan or magnetic resonance imaging in a larger population would be recommended.
