# Supplementary material for: The Influence of Tunnel Parameters and Graft Inclination Angle on Clinical and Radiological Outcome at Long-term Follow-up after Arthroscopic Anterior Cruciate Ligament Reconstruction
Source: Rev Bras Ortop (Sao Paulo). 2024 Apr 10;59(2):e189–98. doi: 10.1055/s-0044-1785493 (PMC11006531; doi:10.1055/s-0044-1785493)
Supplement: Related texts

# Research article: Influência dos parâmetros do túnel e do ângulo de inclinação do enxerto no desfecho clínico e radiológico no acompanhamento de longo prazo após a reconstrução artroscópica do ligamento cruzado anterior

**Keywords:** articulação do joelho, osteoartrite, reconstrução do ligamento cruzado anterior

## Abstract

**Objetivo** O objetivo deste estudo foi analisar a influência de vários parâmetros do túnel e do ângulo de inclinação do enxerto (GIA, do inglês *graft inclination angle* ) nos desfechos clínicos e radiológicos da reconstrução do ligamento cruzado anterior (RLCA) no acompanhamento de longo prazo.

**Métodos** Neste estudo retrospectivo, 80 pacientes com lesão isolada do ligamento cruzado anterior (LCA) submetidos à RLCA de feixe único com autoenxertos de tendão patelar ósseo (TPO) e isquiotibiais (IT) foram avaliados clínica e radiologicamente durante o acompanhamento em longo prazo. A população do estudo foi dividida em dois grupos com base nos parâmetros ideais ou não ideais do túnel, bem como no GIA ideal e não ideal. Os vários parâmetros do túnel e o GIA foram interpretados com os desfechos clínicos e radiológicos no acompanhamento em longo prazo.

**Resultados** Oitenta pacientes, sendo 36 (45%) submetidos ao procedimento com autoenxertos de TPO e 44 (55%) com autoenxertos IT, puderam completar o estudo. Pacientes com ângulo do túnel tibial coronal (ATTC) e ângulo do túnel femoral coronal (ATFC) ideais apresentam resultados clínicos superiores (teste de *pivot shift* ) do que aqueles com ATTC e ATFC não ideais, sendo a diferença estatisticamente significativa (valor de *p* < 0,038 e 0,024, respectivamente). Da mesma forma, pacientes com posição do túnel tibial coronal (PTTC) ideal apresentam resultado clínico superior (International Knee Documentation Committee [IKDC] objetivo) em relação àqueles com PTTC não ideal (valor de *p* < 0,017). Os demais parâmetros do túnel e o GIA não influenciaram o desfecho clínico. Nenhum dos parâmetros do túnel influenciou a alteração associada à osteoartrite (OA). Não houve progressão da alteração da OA na população do estudo no acompanhamento em longo prazo após a RLCA.

**Conclusão** Os parâmetros ideais do túnel coronal produziram um melhor desfecho clínico no acompanhamento de longo prazo após a RLCA. Não houve progressão da alteração da OA no acompanhamento em longo prazo após a RLCA isolada.

## Introdução

A reconstrução artroscópica do ligamento cruzado anterior (RLCA) é o padrão terapêutico para a insuficiência do ligamento cruzado anterior (LCA). O objetivo da RLCA é restaurar a anatomia e cinesiologia normais do joelho, o que melhora a estabilidade da articulação. Segundo a literatura recente, alterações precoces associadas à osteoartrite (OA) podem ser abortadas no joelho com lesão de LCA pela RLCA anatômica. 1 As colocações dos túneis tibial e femoral são bastante importantes para a estabilidade do joelho. A colocação inadequada do túnel femoral ou tibial foi a causa mais comumente discutida para a insuficiência do LCA. 2 Estudos cadavéricos mostraram que, em radiografias laterais, a localização do centro do LCA nativo no fêmur é observada em uma média de 66% da borda anterior da linha de Blumensaat. No lado tibial, o centro do LCA está na junção do terço anterior e médio do platô tibial. 3 Apoiando os estudos cadavéricos, estudos clínicos recentes mostram que a RLCA anatômica dá melhor resultado clínico do que a reconstrução não anatômica. 4 A técnica transtibial (TT) tradicional levaria à orientação mais vertical do enxerto, diminuição da estabilidade rotacional e falha do enxerto. 5 Argumentou-se que o portal anteromedial (AM) auxilia a colocação mais anatômica do túnel femoral em comparação ao portal TT tradicional. Os enxertos no LCA anatômico ficam em uma posição mais horizontal no plano coronal, melhorando a estabilidade rotacional e diminuindo o fenômeno de *pivot shift.* 6 Embora os parâmetros ideais do túnel e o ângulo de inclinação do enxerto (GIA, do inglês *graft inclination angle* ) representando o LCA anatômico tenham sido enfatizados em muitos estudos anteriores, sua influência nos resultados em longo prazo não foi validada. Além disso, poucos autores acreditam que não há correlação entre a posição do túnel e o resultado clínico em longo prazo. 7 Ademais, estudos recentes mostraram que a colocação não anatômica do túnel femoral tem a mesma prevalência entre as técnicas com portais TT e AM. 8 Apesar da extensa literatura sobre a colocação do túnel, o método ideal não foi determinado em muitos pacientes devido a algumas variáveis. Dentre os parâmetros do túnel comumente usados, estão posição do túnel femoral coronal (CFTP, do inglês *coronal femoral tunnel position* ), posição do túnel femoral sagital (SFTP, do inglês *sagittal femoral tunnel position* ), posição do túnel tibial coronal (CTTP, do inglês *coronal tibial tunnel position* ), posição do túnel tibial sagital (STTP, do inglês *sagittal tibial tunnel position* ), ângulo do túnel femoral coronal (CFTA, do inglês *coronal femoral tunnel angle* ), ângulo do túnel femoral sagital (SFTA, do inglês *sagittal femoral tunnel angle* ), ângulo do túnel coronal tibial (CTTA, do inglês *coronal tibial tunnel angle* ), ângulo sagital do túnel tibial (STTA, do inglês *sagittal tibial tunnel angle* ) e GIA.

Portanto, propusemos estudar retrospectivamente a influência de vários parâmetros do túnel e GIA nos desfechos clínicos e radiológicos após a RLCA no acompanhamento em longo prazo.

Nossas hipóteses são:

1. Os parâmetros ideais do túnel coronal levariam a um melhor desfecho clínico.
2. A progressão da OA do joelho seria retardada pela RLCA em casos de lesão isolada do LCA.

## Materiais e métodos

Este estudo retrospectivo foi realizado em um centro de atendimento terciário no sul da Índia. O estudo foi aprovado pelo Comitê de Ética e todos os pacientes do estudo assinaram o termo de consentimento livre e esclarecido.

De janeiro de 2013 a agosto de 2016, foram coletados dados de 80 pacientes do registro hospitalar, compreendendo 36 pacientes submetidos a RLCA com autoenxerto de tendão patelar ósseo (TPO) e 44 pacientes com autoenxertos de isquiotibiais (IT).

Critérios de inclusão:

Idade > 18 anos e < 45 anos

• Sexo masculino e feminino
• Lesão isolada do LCA
• Disponibilidade para participação no estudo
Critérios de exclusão:
• Lesões meniscais e condrais associadas
• Lesão multiligamentar
• Cirurgias anteriores em membro ou joelho ipsilateral
• Fraturas concomitantes no membro ipsilateral ou contralateral
• Nova lesão no joelho operado
• Pacientes com lassidão ligamentar generalizada
• Pacientes que não desejavam participar do estudo

## Procedimento cirúrgico

Todos os pacientes deste estudo foram submetidos a RLCA artroscópica por um único cirurgião artroscópico treinado. A técnica de portal AM foi utilizada em todos os casos para confecção do túnel femoral. Os pacientes receberam autoenxerto de TPO ou IT. A fixação do enxerto lateral femoral e tibial foi feita com parafuso de interferência de titânio (Nebula, Índia).

Reabilitação pós-operatória:

**Table:** 

Semana 1	Isometria de quadríceps, bombas de tornozelo, exercício ativo de elevação da perna esticada (do inglês, *straight leg raise* [SLR]) com joelheira.
Semana 2	Exercício anterior + mobilização patelar e exercício de amplitude de movimento do joelho em cadeia fechada restringindo a flexão a 90 graus.
Semana 3–6	Exercício anterior + exercício progressivo de fortalecimento de isquiotibiais e quadríceps.
Semana 6–3 meses	Exercício anterior + flexão progressiva do joelho + exercícios de propriocepção e fortalecimento da musculatura abdominal, lombar, pélvica e do quadril ( *core* ).
3–6 meses	Natação, agachamento progressivo e exercícios de treinamento de agilidade específicos para esportes.

## Avaliação radiológica

Radiografias simples pré-operatórias do joelho lesionado foram comparadas às radiografias pós-operatórias. As radiografias analisadas eram em projeção anteroposterior (AP) em pé com 0° de flexão de joelho, posteroanterior (PA) com 30° de flexão de joelho e lateral com 30° flexão de joelho. Todas as imagens radiográficas foram interpretadas em formato DICOM em PACS (versão 8.2). Os diversos parâmetros do túnel e as medidas de alterações GIA e OA foram analisados. Os parâmetros do túnel e as medidas radiológicas analisadas no estudo foram CFTA, SFTA, CTTA, STTA, CFTP, SFTP, CTTP, STTP e GIA ( Figs. 1 e 2 ). A análise de alterações da AO foi baseada nos escores de Kellegran e Lawrence (KL). 9 A avaliação radiológica foi feita por dois radiologistas com correlações interobservador e intraobservador confiáveis.

**Fig. 1:** Avaliação radiológica dos ângulos dos túneis em radiografias pós-operatórias. A) Ângulos coronais do túnel femoral e tibial. B) Ângulos sagitais do túnel femoral e tibial. C) Ângulo de inclinação do enxerto.

**Fig. 2:** Avaliação radiológica das posições dos túneis em radiografias pós-operatórias. A) Posições coronais dos túneis femoral e tibial. B) Posições sagitais dos túneis femoral e tibial.

## Avaliação clínica

A avaliação clínica incluiu o *International Knee Documentation Committee* (IKDC) subjetivo e objetivo, o escore de Lysholm, teste de salto unipodal (TSU), teste de *pivot shift* , teste de gaveta anterior (TGA) e teste de Lachman (TL).

A população do estudo foi dividida em dois grupos com base nos parâmetros ideais ou não do túnel e GIA ideal ou não ideal.

Os parâmetros ideais do túnel e o GIA ideal foram aqueles em que as medidas estavam dentro do intervalo de referência recomendado por estudos anteriores. 10 11 12 13 14 Os parâmetros não ideais e GIA não ideal foram aqueles em que as medidas estavam fora do intervalo de referência recomendado por estudos anteriores ( Figs. 3 e 4 ). O teste de *pivot shift* foi dicotomizado em 2 subgrupos para análise estatística, sendo um subgrupo de grau 0 e outro com graus 1, 2 e 3. Da mesma forma, TGA e TL foram dicotomizados em 2 grupos, sendo um de grau 1 e outro de graus 2 e 3. Da mesma forma, o IKDC (objetivo) e o teste de salto unipodal foram dicotomizados em grupos de graus A e B e graus C e D.

**Fig. 3:** Diagrama esquemático mostrando a faixa ideal de ângulo do túnel. A) Faixa ideal de ângulo do túnel coronal femoral e tibial. B) Faixa ideal de ângulo sagital do túnel femoral e tibial. C) Faixa ideal de ângulo de inclinação do enxerto.

**Fig. 4:** Diagrama esquemático mostrando a faixa ideal de posição do túnel. A) Faixa ideal de posição coronal do túnel femoral e tibial. B) Faixa ideal de posição sagital do túnel femoral e tibial.

Os desfechos clínicos e radiológicos foram interpretados nos grupos com túneis ideais e não ideais, bem como nos grupos de GIA ideais e não ideais no acompanhamento em longo prazo.

## Análise estatística

A análise estatística foi feita com o IBM-SPSS Statistics for Windows versão 26.0 (IBM Corp., Armonk, NY, EUA). Dados descritivos como média, mediana e desvio-padrão (DP) foram inseridos como números e porcentagens. A estatística analítica foi realizada por meio do teste qui-quadrado, teste de Mann-Whitney e teste T pareado após a dicotomização. O valor de *p* foi significativo se < 0,05.

## Resultados

Oitenta pacientes, sendo 36 (45%) com autoenxerto de TPO e 44 (55%) com autoenxerto de IT, foram incluídos no estudo. A média (DP) de idade dos pacientes foi de 31,25 (6,83). Cerca de 34 (42,5%), 32 (40%) e 14 (17,5%) dos pacientes pertenciam à faixa etária de 20 a 29 anos, 30 a 39 anos e 40 a 49 anos, respectivamente. Havia 71 (88,8%) pacientes do sexo masculino e 9 (11,3%) pacientes do sexo feminino. Acidentes de trânsito (34 casos [42,5%]), queda de própria altura (24 [30%]) e lesões esportivas (22 [27,5%]) foram relatados como os diferentes modos de lesão. O tempo mediano (intervalo interquartil, IIQ) desde a lesão foi de 90 (30–180) dias. O tempo médio (DP) de acompanhamento dos pacientes foi de 98,59 (+/- 13,78) meses. A distribuição dos parâmetros ideais/não ideais do túnel e GIA ideal/não ideal entre os participantes do estudo é mostrada nas Figs. 5 6 7 .

**Fig. 5:** Distribuição do parâmetro ideal/não ideal do túnel tibial na população estudada. CTTA, Ângulo do túnel tibial coronal; STTA, ângulo sagital do túnel tibial; CTTP, posição do túnel tibial coronal; STTP, posição do túnel tibial sagital.

**Fig. 6:** Distribuição dos parâmetros ideais/não ideais do túnel femoral entre na população estudada. CFTA, Ângulo do túnel femoral coronal; SFTA, ângulo do túnel femoral sagital; CFTP, posição do túnel femoral coronal; SFTP, posição do túnel femoral sagital.

**Fig. 7:** Distribuição do ângulo de inclinação do enxerto (GIA ideal/não ideal) entre a população estudada.

A média dos parâmetros ideais do túnel e do GIA ideal em nosso estudo foi comparada aos valores de referência da literatura, como mostra a Tabela 1 .

**Tabela 1:** Comparação da média dos parâmetros do túnel e ângulo de inclinação do enxerto (GIA) do nosso estudo com valores da literatura

Parâmetros do túnel	Nosso estudo média (DP)	Valor de referência média (DP)	Literatura
CFTA	35,13 (7,61)	39,5 (6,5)	Illingworth et al. (2011)
SFTA	23,85 (11,315)	31 (11)	Takeda et al. (2013)
CTTA	19,4 (8,824)	28 (18)	Kondo et al. (2007)
STTA	31,33 (7,202)	69,5 (19,5)	Kondo et al. (2007)
CFTP	41,76 (3,135)	42 (5)	Pinczewski et al. (2008)
SFTP	59,14 (16,834)	86 (10)	Pinczewski et al. (2008)
CTTP	44,86 (4,469)	46 (5)	Pinczewski et al. (2008)
STTP	37,98 (8,35)	48 (5)	Pinczewski et al. (2008)
GIA	15,81 (7,621)	19 (5)	Pinczewski et al. (2008)

Abreviaturas: DP, desvio-padrão; CFTA, ângulo do túnel femoral coronal; SFTA, ângulo do túnel femoral sagital; CTTA, ângulo do túnel coronal tibial; STTA, ângulo sagital do túnel tibial; CFTP, posição do túnel femoral coronal; SFTP, posição do túnel femoral sagital; CTTP, posição do túnel tibial coronal; STTP, posição do túnel tibial sagital; AIE, ângulo de inclinação do enxerto.

Em relação ao desfecho clínico subjetivo, as médias (DP) pré e pós-operatórias do escore IKDC (subjetivo) foram 49,15 (9,86) e 91,10 (6,97), enquanto as médias (DP) pré e pós-operatórias do escore Lysholm foram de 38,30 (11,52) e 94,06 (4,81), respectivamente. Não houve diferença significativa entre os parâmetros ideais e não ideais do túnel e GIA em relação ao desfecho clínico subjetivo.

A distribuição dos resultados clínicos e radiológicos nos pacientes do estudo é mostrada na Tabela 2 . O escore LK atual pós-operatório é igual aos escores LK pré-operatórios na população do estudo. A associação estatística entre vários parâmetros do túnel e o desfecho clínico (objetivo) é mostrada na Tabela 3 . A Tabela 4 mostra a ausência de diferenças significativas entre o GIA ideal e não ideal e o desfecho clínico (objetivo) e radiológico. Da mesma forma, não houve diferença significativa entre outros parâmetros do túnel no desfecho radiológico, como mostra a Tabela 5 .

**Tabela 2:** Distribuição dos desfechos clínicos e radiológicos nos pacientes do estudo

Parâmetros de desfechos clínicos objetivos	Valor pré-operatório N (%)	Valor atual pós-operatório N (%)
**Teste de** ***pivot shift***		
Grau 0	0 (0)	36 (45)
Grau 1	2 (2,5)	42 (52)
Grau 2	50 (62,5)	2 (2,5)
Grau 3	28 (35)	0 (0)
**Escore IKDC subjetivo**		
A	0 (0)	48 (60)
B	0 (0)	31 (38,8)
C	74 (92,5)	1 (1,3)
D	6 (7,5)	0 (0)
**Grau IKDC do teste de salto unipodal**		
A	0 (0)	62 (77,5)
B	0 (0)	15 (18,8)
C	0 (0)	3 (3,8)
**Teste da gaveta anterior**		
Grau 1	0 (0)	37 (46,3)
Grau 2	34 (42,5)	39 (48,8)
Grau 3	46 (57,5)	4 (5)
**Teste de Lachman**		
Grau 1	0 (0)	36 (45)
Grau 2	30 (37,5)	42 (52,5)
Grau 3	50 (62,5)	2 (2,5)
**Escore KL**		
Grau 1	22 (27,5)	22 (27,5)
Grau 2	6 (7,5)	6 (7,5)
Normal	52 (65)	52 (65)
**Escore IKDC (subjetivo)**		
Média	49,499	91,067
Valor mínimo	6,9	66,7
Valor máximo	71,3	98,9
Desvio-padrão	13,856	6,099
**Escore de Lysholm**		
Média	38,35	94,05
Valor mínimo	2	80
Valor máximo	66	100
Desvio-padrão	11,584	4,846

Abreviaturas: IKDC, *International Knee Documentation Committee* ; KL, Kellegran e Lawrence.

**Tabela 3:** Associação estatística dentre diversos parâmetros do túnel e desfechos clínicos expressa como valor de *p*

Ideal *vs* não ideal	Teste de salto unipodal	IKDC (objetivo)	Teste de *pivot shift*	Teste de gaveta anterior	Teste de Lachman
CTTA	0,458	0,672	0,038*	0,363	0,766
STTA	0,527	0,302	0,514	0,666	0,514
CTTP	0,365	0,017*	0,378	0,730	0,801
STTP	0,062	0,062	0,127	0,080	0,095
CFTA	0,975	0,147	0,024*	0,332	0,737
SFTA	0,858	0,113	0,503	0,191	0,096
CFTP	0,556	0,737	0,294	0,331	0,294
SFTP	0,365	0,672	0,378	0,109	0,378

Abreviaturas: CFTA, ângulo do túnel femoral coronal; SFTA, ângulo do túnel femoral sagital; CTTA, ângulo do túnel tibial coronal; STTA, ângulo sagital do túnel tibial; CFTP, posição do túnel femoral coronal; SFTP, posição do túnel femoral sagital; CTTP, posição do túnel tibial coronal; STTP, posição do túnel tibial sagital; GIA, ângulo de inclinação do enxerto; IKDC, *International Knee Documentation Committee* .

*- indica valor de *p* < 0,05.

**Tabela 4:** Associação entre o ângulo de inclinação do enxerto (GIA) e desfecho

Ideal *vs* não ideal	Teste de salto unipodal	Escore KL	IKDC	Teste de *pivot shift*	Teste de gaveta anterior	Teste de Lachman
GIA (valor de *p* )	0,325	0,672	0,574	0,196	0,066	0,068

Abreviaturas: GIA, Ângulo de inclinação do enxerto; ESCORE KL, ESCORE de Kellgren e LawrenceI; KDC, Comitê Internacional de Documentação do Joelho; ADT, Teste da Gaveta Anterior; LT, Teste de Lachman.

**Tabela 5:** Associação entre os parâmetros do túnel e o desfecho radiológico

Ideal vs. Não ideal	CTTA	STTA	CTTP	STTP	CFTA	SFTA	CFTP	SFTP
Escore KL (valor de *p* )	0,905	0,078	0,285	0,093	0,341	0,796	0,058	0,905

Abreviaturas: KL SCORE, ESCORE de Kellgren e Lawrence; CFTA, ângulo do túnel femoral coronal; SFTA, ângulo sagital do túnel femoral; CTTA, ângulo coronal do túnel tibial; STTA, ângulo sagital do túnel tibial; CFTP, posição do túnel femoral coronal; SFTP, posição sagital do túnel femoral; CTTP, posição coronal do túnel tibial; STTP, posição sagital do túnel tibial.

Pacientes com CTTA e CFTA ideais apresentaram resultado clínico estatisticamente superior (teste de *pivot shift* ) do que aqueles com CTTA e CFTA não ideais (valor de *p* < 0,038 e 0,024, respectivamente). Da mesma forma, os pacientes com CTTP ideal apresentaram desfecho clínico superior (IKDC objetivo) em relação aos com CTTP não ideal (valor de *p* < 0,017), conforme mostrado na Tabela 6 .

**Tabela 6:** Parâmetros ideais do túnel coronal mostrando a superioridade estatisticamente significativa dos desfechos clínicos em relação aos parâmetros não ideais do túnel coronal

Parâmetros do túnel		Desfecho clínico (teste de *pivot shift* )		Qui-quadrado (valor de *p* )
		Grau 0	Graus 1, 2 e 3	
**CTTA**	Ideal	31	29	0,038
	Não ideal	5	15	
**CFTA**	Ideal	29	25	0,024
	Não ideal	7	19	
		**Desfecho clínico** **(IKDC objetivo)**		
		**Graus A e B**	**Graus C e D**	
**CTTP**	Ideal	68	0	0,017
	Não ideal	11	1	

Abreviaturas: CTTA, ângulo do túnel tibial coronal; CFTA, ângulo do túnel femoral coronal; CTTP, posição do túnel tibial coronal; gr, grau.

## Discussão

O principal achado de nosso estudo é que pacientes com CFTA e CTTA ideais apresentaram melhor estabilidade rotacional, com diminuição do grau de *pivot shift* . Isso implica que CFTA e CTTA ideais, que produziriam túneis femorais e tibiais mais oblíquos, melhoram a estabilidade rotacional. Da mesma forma, pacientes com CTTP ideal têm melhor pontuação no escore IKDC (objetivo). Outros parâmetros do túnel e o GIA não tiveram qualquer impacto significativo no desfecho clínico.

Portanto, nossa primeira hipótese é comprovadamente correta, pois os parâmetros ideais do túnel coronal melhoraram os desfechos clínicos ao melhorarem de forma significativa o grau de *pivot shift* e o escore IKDC (objetivo). Da mesma forma, nossa segunda hipótese também se provou correta, pois não houve progressão da OA do joelho após a RLCA isolada na população do estudo durante o acompanhamento em longo prazo. Nosso estudo mostra claramente que a colocação coronal precisa dos enxertos nos lados femorais e tibiais é essencial para a obtenção de melhores desfechos clínicos em longo prazo após a RLCA.

De acordo com diversos estudos, a colocação do enxerto mais verticalmente no plano coronal faria com que o enxerto colidisse com a parte lateral do ligamento cruzado posterior (LCP), causando perda de flexão, diminuição da estabilidade anterior e, por consequência, mau desfecho clínico. 5 11 15 16 A colocação mais oblíqua do túnel femoral no plano coronal é importante para a estabilidade rotacional do joelho. 10

Embora se acreditasse que a técnica com portal AM auxiliaria a posição baixa e a obliquidade do CFTA, ainda há controvérsias quanto à sua superioridade sobre a técnica com TT. Ruhr-Wagner et al. 17 relataram maior risco de cirurgias de revisão com a técnica de portal AM em comparação à técnica de TT. Da mesma forma, Jaecker et al. 8 observaram altas taxas de posições não anatômicas dos túneis femoral e tibial em revisões de cirurgias do LCA com técnicas de perfuração femoral AM e TT. Além disso, em nosso estudo, utilizamos apenas a técnica de portal AM para perfuração femoral em todos os casos. Encontramos posições e ângulos não anatômicos dos túneis em muitos de nossos pacientes. Isto pode ser atribuído a variações anatômicas femorais distais individuais, como a incisura femoral estreita, que pode não permitir a perfuração oblíqua do túnel; isso pode fazer com que um túnel não anatômico seja colocado anteriormente com CFTA menor, como postulado por Illingworth et al. 11

Em nosso estudo, o CFTA médio foi de 35,13°. Illingworth et al. 11 avaliaram o CFTA em radiografias PA com carga e peso de flexão do joelho de 45° em pacientes com RLCA no período pós-operatório e descobriram que o CFTA < 32,7° provavelmente foi submetido a uma RLCA fora da faixa anatômica. Em nosso estudo, os pacientes com ângulos não ideais do túnel femoral, < 32°, apresentaram maior grau de *pivot shift* no período pós-operatório. Isso contrasta com os estudos de Sundemo et al. 7 e Moghtadaei et al. 18 , que não encontraram influência do CFTA no desfecho clínico ou radiológico. Em nosso estudo, observamos que pacientes com CFTA ideal apresentaram grau de *pivot shift* significativamente melhor em comparação àqueles com CFTA não ideal. Em nosso estudo, o CTTA médio foi de 19,40°. O CTTA ideal descrito na literatura varia entre 60 e 65° (medido entre o platô tibial e o túnel). 19 Isso evita o impacto do LCP e diminui a lassidão anterior. Em nosso estudo, o CTTA foi medido entre o eixo anatômico da tíbia e o túnel tibial, como descrito por Kondo et al. 13 Uma medida semelhante foi feita por Moghtadaei et al. 18 Em seu estudo, o CTTA não influenciou o desfecho da RLCA. Em nosso estudo, descobrimos que os pacientes com CTTA ideal tinham estabilidade rotacional significativamente melhor do que aqueles com CTTA não ideal. De acordo com Pinczewski et al. 10 e Topliss e Webb, 20 o túnel tibial deve estar a 47% do córtex medial através do planalto tibial no plano coronal. Estes autores afirmaram que uma colocação mais medial pode causar impacto. Em nosso estudo, o CTTP médio foi de 44,86° (+/- 4,46). Os pacientes com CTTP ideal em nosso estudo tiveram melhor escore IKDC (objetivo) do que aqueles com CTTP não ideal.

Debnath et al. 21 fizeram uma avaliação radiológica da posição do túnel na RCLA de feixe único na população indiana e a correlacionaram ao desfecho clínico. Estes autores descobriram que o “desfecho clínico ideal” estava significativamente associado à colocação do túnel femoral ao longo do plano sagital. Também recomendam que o túnel femoral não deva ser colocado além da marca de 85% ao longo da linha Blumensat a partir do ponto mais anterior. Xu et al., 22 em uma revisão sistemática, relataram que a posição média da inserção femoral nativa foi de 28,4% (+/- 5,1%) da borda posterior com a utilização do método do quadrante. Sundemo et al. 7 relataram que a SFTP média pelo método quadrante foi de 40% (+/- 6,4%) de posterior a anterior. Em nosso estudo, a SFTP média foi de 59,14 (+/- 16,83). Não observamos a influência de SFTP em desfechos clínicos ou radiológicos. De acordo com Ristić et al., 14 o STTA aceitável foi de 50 a 89° (média de 68°). Estes autores relatam que um desvio significativo desses valores de pode levar ao insucesso da RLCA. Em nosso estudo, o STTA médio foi 31,33° (+/- 7,20). Não houve diferença nos desfechos com STTA ideal e não ideal em nosso estudo.

Moisala et al. 23 afirmaram que a localização ideal do túnel tibial no plano sagital é entre 32 e 37% do comprimento do platô tibial a partir do canto anterior para melhor desfecho clínico. Em nosso estudo, a STTP foi de 37,98 (+/- 8,35). Não observamos qualquer influência da STTP no desfecho clínico.

Analisamos a influência de diversos parâmetros do túnel e do GIA no desfecho da RLCA com os dois autoenxertos comumente usados. Poucos autores fizeram estudos semelhantes sobre os parâmetros do túnel utilizando esses dois enxertos, com resultados variáveis. 7 24 25

Pinczewski et al. 10 e Struewer et al. 26 relataram a progressão de OA em sua população de estudo após a RLCA em longo prazo. Ao contrário destes autores, não observamos influência dos parâmetros do túnel ou GIA nas alterações em longo prazo da OA. Nossos resultados foram semelhantes aos de Sundemo et al. 7 Surpreendentemente, nosso estudo revelou que pacientes com OA pré-existente também não apresentaram piora em longo prazo. Isso implica que além de parâmetros do túnel e GIA, fatores como lesões meniscais ou condrais concomitantes podem ser importantes contribuintes na progressão em longo prazo das alterações da OA após a RLCA.

## Limitações

Nosso estudo tem algumas limitações. Realizamos o estudo apenas em pacientes isolados com LCA. Para que a população de estudo fosse adequada, a inclusão de dois tipos de autoenxertos era inevitável. Embora isso tenha tornado a população do estudo heterogênea, não influenciou o resultado. Por ser um estudo retrospectivo, um possível viés de seleção foi inevitável. Usamos apenas radiografia simples para avaliar os parâmetros do túnel. A classificação dos testes anteroposterior e *pivot shift* foi realizada manualmente em vez de utilizar artrômetros instrumentados.

Este estudo foi único na medida em que analisamos a influência de até nove parâmetros relacionados ao túnel, que incluem posições do túnel femoral e tibial, ângulos do túnel e GIA no desfecho clínico e radiológico em longo prazo, o que torna o nosso estudo distinguível dos demais.

## Conclusão

Em nosso estudo, pacientes com parâmetros ideais do túnel coronal apresentaram estabilidade rotacional e desfecho clínico significativamente melhores em comparação àqueles com parâmetros não ideais do túnel no acompanhamento em longo prazo após a RLCA. Os parâmetros sagitais do túnel e o GIA não influenciaram de forma significativa os desfechos clínicos ou radiológicos após a RLCA. Nenhum dos parâmetros do túnel ou GIA teve influência nas alterações da OA em longo prazo. Recomendamos a realização de estudos prospectivos futuros comparando desfechos clínicos e radiológicos em longo prazo após a RLCA usando tomografia computadorizada tridimensional (3D) ou ressonância magnética em uma população maior.
